# Where does *Neisseria* acquire foreign DNA from: an examination of the source of genomic and pathogenic islands and the evolution of the *Neisseria* genus

**DOI:** 10.1186/1471-2148-13-184

**Published:** 2013-09-04

**Authors:** Catherine Putonti, Bogdan Nowicki, Michael Shaffer, Yuriy Fofanov, Stella Nowicki

**Affiliations:** 1 Department of Biology, Loyola University Chicago, 1032 W. Sheridan Rd, Chicago, IL 60660, USA; 2 Department of Computer Science, Loyola University Chicago, Chicago, IL 60660, USA; 3 Bioinformatics Program, Loyola University Chicago, Chicago, IL 60626, USA; 4 Department of Obstetrics& Gynecology, Meharry Medical College, Nashville, TN 37208, USA; 5 Department of Computer Science, University of Houston, Houston, TX 77204, USA; 6 Department of Biology and Biochemistry, University of Houston, Houston, TX 77204, USA

**Keywords:** *Neisseria*, Horizontal gene transfer, Pathogenicity islands, Pathogen-host DNA transfer

## Abstract

**Background:**

Pathogenicity islands (PAIs) or genomic islands (GEIs) are considered to be the result of a recent horizontal transfer. Detecting PAIs/GEIs as well as their putative source can provide insight into the organism’s pathogenicity within its host. Previously we introduced a tool called S-plot which provides a visual representation of the variation in compositional properties across and between genomic sequences. Utilizing S-plot and new functionality developed here, we examined 18 publicly available *Neisseria* genomes, including strains of both pathogenic and non-pathogenic species, in order to identify regions of unusual compositional properties (RUCPs) using both a sliding window as well as a gene-by-gene approach.

**Results:**

Numerous GEIs and PAIs were identified including virulence genes previously found within the pathogenic *Neisseria* species. While some genes were conserved amongst all species, only pathogenic species, or an individual species, a number of genes were detected that are unique to an individual strain. While the majority of such genes have an origin unknown, a number of putative sources including pathogenic and capsule-containing bacteria were determined, indicative of gene exchange between *Neisseria* spp. and other bacteria within their microhabitat. Furthermore, we uncovered evidence that both *N. meningitidis* and *N. gonorrhoeae* have separately acquired DNA from their human host. Data suggests that all three *Neisseria* species have received horizontally transferred elements post-speciation.

**Conclusions:**

Using this approach, we were able to not only find previously identified regions of virulence but also new regions which may be contributing to the virulence of the species. This comparative analysis provides a means for tracing the evolutionary history of the acquisition of foreign DNA within this genus. Looking specifically at the RUCPs present within the 18 genomes considered, a stronger similarity between *N. meningitidis* and *N. lactamica* is observed, suggesting that *N. meningitidis* arose before *N. gonorrhoeae*.

## Background

Infectious diseases, which remain a major cause of human morbidity/mortality, are the direct result of interactions between the human host and unique sets of microbial virulence factors. These virulence factors have often been found in clusters of horizontally transferred 10–100 kb genomic DNA regions [1-3] or in short scattered virulence-associated 1–10 kb islets [4]. Regions of DNA introduced through horizontal transfer, referred to as genomic islands (GEIs), can encode for genes of a variety of functions. Identification of the subset of GEIs that encode for virulence factors, referred to as pathogenicity islands (PAIs), can lead to the understanding of the organism’s pathogenicity within the host.

There is considerable interest in developing statistical methods to predict PAIs and/or GEIs for future experimental validation as well as furthering our understanding of pathogenicity. As such, numerous statistical methods have been developed to identify aberrant regions in terms of nucleotide composition [5-20] as well as examining phylogenetic discrepancies [21-27]. While alone each of these techniques is appropriate for the detection of a particular GEI/PAI feature, no single approach is capable of detecting all GEIs and PAIs. Combinatorial approaches, e.g. [28-33], although often more cumbersome and inconclusive (in the case where a region is identified by just some of the measures used), can discover more GEIs and PAIs. Beyond just detecting GEIs and PAIs is the task of identifying the source of the genes acquired. This is further complicated when looking at species with open pan-genomes (in which new strains always include novel genes) such as *Neisseria meningitidis*[34-36].

A graphic approach called Similarity Plot or S-plot for rapid large scale comparison, analysis, and visualization of genomic sequences was previously employed to recognize regions within a genomic sequence that originated through horizontal gene transfer as well as genes under specific selective forces [37]. This tool, as described in detail in our previous work [37], identifies regions of unusual compositional properties (RUCPs) via a sliding-window approach. Recently we expanded this functionality, permitting gene-by-gene comparative analyses to be performed.

Herein we present the results of a comparative analysis of all complete annotated *Neisseria* genomes currently available. This includes 18 genomes: three from the species *N*. *gonorrhoeae*, 14 from the species *N. meningitidis* and one from the non-pathogenic species *N*. *lactamica*. As previous analysis has shown, *N. meningitidis* exhibits a moderately strong codon bias effect [38,39]. As such, recently acquired genes will likely exhibit a codon usage more attuned to the tRNA abundances within their native genome. Through the identification of horizontally acquired elements, we can better understand the evolution of this genus with respect to the transformations occurring at the genomic level as well as the variations in virulence between the species and strains. Looking specifically at the RUCPs present within all 18 genomes considered, a stronger similarity between *N. meningitidis* and *N. lactamica* is observed, suggesting that *N. meningitidis* arose before *N. gonorrhoeae* as shown in previous studies [40].

## Results

### Visualizing similarities and dissimilarities in compositional properties within ***Neisseria*** spp

**Figure 1:**
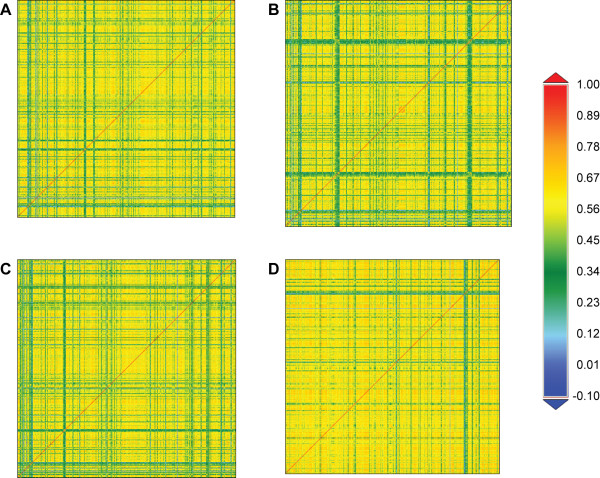
**Four *Neisseria *genomes compared against themselves via S-plot.** The color-coded scale on the right indicates the level of similarity. **(A)** serogroup A *N. meningitidis* Z2491 **(B)** serogroup B *N. meningitidis* MC58, **(C)** serogroup C *N. meningitidis* FAM18, and **(D)***N. gonorrhoeae* FA 1090.

For each of the 18 *Neisseria* strains listed in Table 1, the genomic sequence was compared to itself based upon its frequency distribution of 6-mers within a sliding window of 5,000 nucleotides (Figure 1). (See Methods for discussion regarding the selection of the subsequence size selection.) By comparing a genome to itself, as shown in Figure 1, it is possible to identify windows with a composition varying from the genomic norm (indicated as the blue lines traversing the S-plot) as well as their distribution throughout the genome. Comparisons between different species and strains revealed rearrangements as well as regions present in one genome and absent from another (Additional file 1: Figure S1, Additional file 2: Figure S2, Additional file 3: Figure S3 and Additional file 4: Figure S4). Additional file 5: Figure S5 compares the *N. meningitidis* serogroup C strain FAM18 with (a) an *N. meningitidis* serogroup A strain, (b) an *N. gonorrhoeae* strain, and (c) the *N. lactamica* strain, highlighting the location of genes associated with the capsule; while the *N. meningitidis* strains contain the entire capsule-synthesis (*cps*) gene cluster, *N. gonorrhoeae* and *N. lactamica* genomes do not [41,42].

**Table 1:** **Number of RUCPs identified for each *Neisseria *strain**

**Species/strain**	**Accession**	**Serogroup**	**# wRUCP**	**# gRUCP**
*N. gonorrhoeae* FA 1090	NC_002946	Ng	16	124
*N. gonorrhoeae* NCCP11945	NC_011035	Ng	27	78
*N. gonorrhoeae* TCDC-NG08107	NC_017511	Ng	22	122
*N. meningitidis* Z2491	NC_003116	NmA	21	141
*N. meningitidis* WUE 2594	NC_017512	NmA	10	144
*N. meningitidis* MC58	NC_003112	NmB	26	62
*N. meningitidis* alpha710	NC_017505	NmB	27	126
*N. meningitidis* G2136	NC_017513	NmB	24	146
*N. meningitidis* M01-240149	NC_017514	NmB	25	135
*N. meningitidis* M04-240196	NC_017515	NmB	25	103
*N. meningitidis* H44/76	NC_017516	NmB	24	121
*N. meningitidis* M01-240355	NC_017517	NmB	28	141
*N. meningitidis* NZ-05/33	NC_017518	NmB	24	128
*N. meningitidis* FAM18	NC_008767	NmC	23	131
*N. meningitidis* 053442	NC_010120	NmC	18	82
*N. meningitidis* 8013	NC_017501	NmC	25	116
*N. meningitidis* alpha14	NC_013016	NmCln	27	126
*N. lactamica* ST-640	NC_014752	Nl	22	146
		*Total*:	414	2172

### Recognizing RUCPs

Examination of the 18 *Neisseria* genomes was conducted using both a window-by-window approach, with a window size of 5000 nucleotides, as well as a gene-by-gene approach. (See Methods for a detailed description of each). We refer to the regions exhibiting unusual compositional properties from the window-by-window analysis as wRUCPs and from the gene-by-gene analysis as gRUCPs. Windows/genes can exhibit unusual compositional properties as a result of horizontal gene transfer or specific selective forces. The number of wRUCPs and gRUCPs varied from strain to strain as well as serogroup to serogroup. In total 414 wRUCPs and 2,172 gRUCPs were identified (Table 1). While the *N. meningitidis* WU 2594 strain (serogroup A) isolated from a patient suffering from acute bacterial meningitidis [43] contained the fewest wRUCPs, 144 genes were identified as having unusual compositional properties; analysis of the location of these genes revealed that there were several clusters of adjacent genes. While some of the gRUCPs include genes which have been assigned a particular gene name and/or function, the majority (77%) are annotated as hypothetical proteins. The complete listing of the wRUCPs and gRUCPs can be found in Additional file 6: Table S1 and Additional file 7: Table S2, respectively. Within the 414 windows identified, 715 (32%) of the genes within these windows were also identified by the gene-by-gene analysis, indicative of larger acquisitions of gene clusters within the acquired DNA fragments. Thus, the gene-by-gene analysis provides a finer granularity for detecting regions of interest.

### Identifying gRUCPs shared within the ***Neisseria*** spp

**Figure 2:**
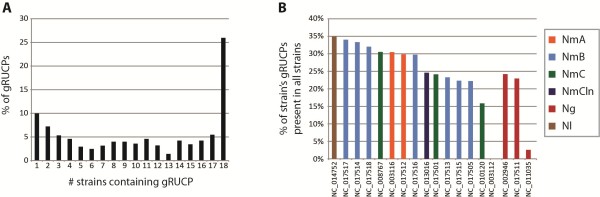
**Examining gRUCPs common between different strains and species of *Neisseria*. (A)** Percentage of gRUCPs identified by the gene-by-gene analysis that are unique to the strain, 1, to present in all strains examined, 18. **(B)** Percentage of each strain’s gRUCPs that are present in all of the *Neisseria* strains examined.

For each gRUCP identified from the gene-by-gene analysis, its frequency profiles of *k*-mers (*k* = 3) were compared to the profiles of all genes within each of the other *Neisseria* strains. (See Methods for a detailed description of *k* selection.) The maximum *R*^2^ was identified for each as a means of quickly recognizing homologous genes. We considered *R*^2^ > 0.9 as likely homologies. As an example, Additional file 8: Figure S6 shows the similarities observed for the gRUCPs within the serogroup A *N. meningitidis* strain Z2491 and the other 17 *Neisseria* genomes. Figure 2 provides a summary of this analysis. Twenty-six percent (564) of the 2,172 gRUCPs identified were common amongst all 18 strains examined. (Note, while some homologous genes were classified as gRUCPs for all 18 strains, others may not have if they did not meet the required similarity threshold.) The percentage of each strain’s gRUCPs that are present in all of the *Neisseria* strains examined varied (Figure 2B). The genes within the non-pathogenic *N. lactamica* genome classified as gRUCPs were frequently found within other *Neisseria* strains; roughly 35% of the gRUCPs had *R*^2^ > 0.9 (Figure 2B) and 65% and 79% of the *N. lactamica* gRUCPs had an *R*^2^ value greater than 0.8 and 0.7, respectively. This is consistent with the notion that a non-pathogenic *Neisseria* species was the ancestor of all extant *Neisseria* species. Given the non-pathogenic nature of *N. lactamica*, genes identified as having unusual compositional properties may be GEIs or genes under different selective pressures and thus exhibiting a composition variant from the norm. In contrast the *N. meningitidis* MC58 (NC_003112) and *N. gonorrhoeae* NCCP11945 (NC_011035) appear to have rather unique gRUCPs; none and only two, respectively, of their gRUCPs are found within all of the other *Neisseria* strains (Figure 2B). While some of the genes in the 18 genomes have been annotated according to their function, many are hypothetical. Additional file 9: Table S3 lists the maximum *R*^2^ for each gRUCP by strain.

Further investigation of the gRUCPs revealed several instances in which RUCPs were found in some but not all strains of the same species. Nevertheless, there were 57 gRUCPs which were conserved amongst all strains of pathogenic *Neisseria* spp., 52 gRUCPs which were conserved amongst only the *N. meningitidis* strains, and 38 gRUCPs which were conserved amongst only the *N. gonorrhoeae* strains. Given the expectation that a homologous gene could be identified as a gRUCP in more than one genome, the sequences for each of the aforementioned gRUCPs were aligned in order to identify the number of unique genes detected as having unusual compositional properties. Each of the homologous gene sequences were then compared to all publicly available bacterial nucleotide and protein sequences using BLASTn and BLASTx. Table 2 summarizes the results of this analysis. On several occasions homologies were found with other *Neisseria* species and strains for which complete genomic sequences are not available. For instance, half of the genes found to be conserved amongst all of the pathogenic *Neisseria* strains and absent from the single *N. lactamica* strain examined here (strain ST-640) BLASTed to the non-pathogenic *N. lactamica* strain 020–06 (whose genome is not yet complete). The similarity between the *N. meningitidis* gRUCP (NMC1806) with annotated FrpC proteins was of interest; this gene has previously been annotated as a PAI [7] and has also been explored as playing a role in meningococcal infections [44,45]. Additional file 10: Table S4, Additional file 11: Table S5, Additional file 12: Table S6, and Additional file 13: Table S7 provide details about the results of the BLAST searches for each of the genes examined.

**Table 2:** **Summary of homologous genes conserved amongst and within only the pathogenic *Neisseria *strains, the *N. meningitidis *strains, and the *N. gonorrhoeae *strains**

**Conserved amongst**	**# Homologous genes**	**Genes found in other genera**
Pathogenic *Neisseria* strains	8	• Putative phage associated protein present in a few *Burkholderia* spp.
		• Annotated in other species, e.g. *Moraxella catarrhalis* and *Haemophilus influenzae*, as ribonuclease T (rnt)
*N. meningitidis* strains	11	• Putative heavy metal transport protein (NMAA_1445)
		• PEMK-like protein
		• Superoxide dismutase*
		• Putative heavy metal transport protein*
		• Hypothetical protein*
		• FrpC protein*
*N. gonorrhoeae* strains	13	• 4 phage-associate proteins
		• Hypothetical proteins *

*Also found in other *Neisseria* species for which complete annotated genomes are not available.

Numerous gRUCPs were present in many but not all of the genomes for a species, indicating strain-specific losses. This was most clearly seen within the *N. mening-itidis* sequences studied. The *N. meningitidis* strains contained 88 gRUCPs that were present within the *N. lactamica* genome and absent from the three *N. gonorrhoeae* genomes. In contrast, there were only four gRUCPs present within the *N. gonorrhoeae* genomes and the *N. lactamica* genome that were not present in any of the *N. meningitidis* strains. Under the premise that all three of these species derived from the same ancestor [46,47], the presence/absence of *N. mening-itidis* and *N. gonorrhoeae* gRUCPs in *N. lactamica* presents insight into their evolutionary history. Either *N. gonorrhoeae* lost the gRUCPs present within the *N. meningitidis* strains or *N. meningitidis* acquired these gRUCPs from *N. lactamica* post-speciation. As previous research has found that there is some genetic exchange between *N. lactamica* and *N. meningitidis*[48,49], the former – the loss of gRUCPs by *N. gonorrhoeae* – is more plausible than the latter.

### PAI presence in RUCPs

Referring to the Virulence Factor Database [50] and previous literature for detecting PAIs within the *Neisseria* spp. [3,7,28,51-54], the RUCPs (both wRUCPs and gRUCPs) were again examined looking for the major virulence factors annotated for *Neisseria*. Genes associated with adherence (primarily the type IV pili), capsule (*siaB*, *siaC*, *siaD*, *synX, lipA, lipB, ctrA, ctrB, ctrC, and ctrD*), as well as genes for invasion (*opa*) were all included in the genes classified as RUCPs. The capsule-related genes were identified as gRUCPs present within *N. meningitidis* and absent from the capsule-lacking *N. gonorrhoeae* strains. Another virulence factor listed in the VFDB is iron uptake as this is a critical function for the survival of *Neisseria* in the host. Genes including the transferrin-binding proteins (*tbpA* and *tbpB*), lactoferrin-binding proteins (*lbpA* and *lbpB*), and ABC-transporters, amongst others, were also identified as RUCPs. Table 3 lists the specific gene IDs for several of the aforementioned known virulence factors found as RUCPs in the *N. meningitidis* strains examined. (See Additional file 6: Table S1 and Additional file 7: Table S2 for additional gene IDs.)

**Table 3:** **Some of the genes which have been previously identified as *Neisseria *virulence factors (VFDB**[50]**) that were found as RUCPs in the *N. meningitidis *strains examined**

**Major virulence factors:**	**Sampling of genes found as RUCPs in *N. meningitidis *strains:**
Adherence (LOS, Type IV pili)	NMA0424
Antiphagocytosis (capsule)	NMBB_0072, NMBB_0073, NMBB_0073A
IgA1 Protease	NMBB_0786
Invasion (Opa, Opc, Porin)	NMBB_1866, NMC1877, NMBNZ0533_1971
Iron uptake (FbpABC, HmbR, HpuAB, Lbp, Tbp)	NMBNZ0533_1791, NMO_1581

Our analysis additionally recognized genes which have been associated with functionality that enables virulence. gRUCPs include the *maf* family of proteins which play a role in adhesion [55] as well as many of the *comE* gene copies which are involved in DNA uptake [56,57]. The RUCPs also contain numerous other proteins involved in the type IV secretion system, including the *tra* family of proteins and *ltgX*, amongst others. T4SS genes are often identified by their homology to the transfer genes of conjugative plasmids or the Ti plasmid of *Agrobac-terium tumefaciens*[58]. The gene *atlA* is classified as a RUCP; this gene has been found to play a critical role in bacterial resistance to phagocytosis and survival in the bloodstream in *Streptococcus mutans*[59]. Providing similar protection from phagocytosis [60], several of the RUCPs are or include genes annotated as Cu-Zn superoxide dismutases (Additional file 6: Table S1 and Additional file 7: Table S2).

### Ascertaining the acquisition and putative sources for gRUCPs

Of the gRUCPs found, 217 appear to be unique to a single *Neisseria* strain (*R*^2^ > 0.9): 80 in *N. gonorrhoeae* strains, 99 in *N. meningitidis* strains, and 38 in the *N. lactamica* genome. The nucleotide sequence of each was then BLASTed against the nr/nt Nucleotide database in an effort to find the putative source of these genes. Of the 217 genes, only 84 (17 *N. gonorrhoeae*, 60 *N. meningitidis* and 7 *N. lactamica*) produced significant similarities (Additional file 13: Table S7). (As a result of these BLAST searches, four were found to be annotated in other genera as 50S ribosomal protein L36, although not in *Neisseria*, and thus removed from further analysis.) In total, 80 of these 213 genes identified as exhibiting unusual compositional properties and unique to a single *Neisseria* strain are present in the genomes of other organisms. Thus, these genes could either be acquired via horizontal gene transfer or their unusual compositional properties are the result of locus specific selective forces, also at play in other bacterial genomes. The remaining 64% of these genes do not show significant sequence similarity to any sequenced species in NCBI’s nucleotide collection.

The BLAST hits were comprised of several hypothetical proteins as well as putative phage associated proteins from a wide variety of bacteria. Homologs to other bacterial species were also identified with annotations including: Cu-Zn superoxide dismutase, excinuclease, chaperone protein DnaK, SecY subunit, zinc transporter, and cation efflux protein. Several of these hits were to other human bacterial pathogens, e.g. *Escherichia coli*, *Helicobacter cinaedi*, *Salmonella enterica*, *Haemophilus influenzae*, *Bordetella pertussis*, *Pseudomonas aeruginosa* and *Rickettsia*, amongst others (Additional file 13: Table S7). Moreover, many of the BLASTn hits of the *N. meningitidis* gRUCPs were to bacteria containing a capsule, including *Pseudomonas* spp., *Kiebsiella* spp., *Haemophilus* spp., *Escherichia* spp., *Salmonella* spp. and *Bordetella* spp.

**Figure 3:**
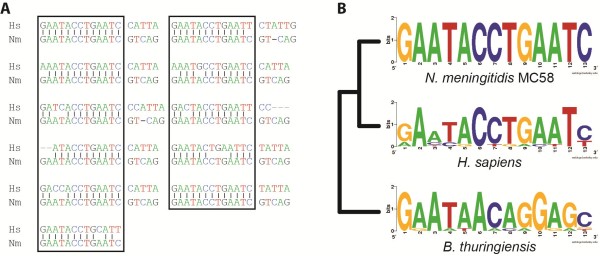
**Repetitive sequence within NMB1848. (A)** Sequence similarity between the NMB1848 sequence (Nm) and the human (Hs) sequence. The repeats found in these two sequences are included within the boxed region. **(B)** Sequence similarity between the consensus of the repeating sequences in *N. meningitidis* NMB1848, human, and *B. thuringiensis.* (Logos and consensus sequences created using WebLogo [62].)

In addition to revealing homologies with sequenced bacteria, two of the gRUCPs were found to share statistically significant similarity with sequences from eukaryotic species. One gRUCP (NMB1848), from the serogroup B strain *N. meningitidis* MC58, showed similarity to human and zebrafish sequences. Homology between NMB1848 and a portion of the PHD finger protein sequence PHF21B was detected in both eukaryotic species. The GO functionality associated with PHF21B is zinc ion binding (Additional file 13: Table S7). The *N. meningitidis* sequence exhibits the greatest sequence similarity with these and other eukaryotic species through a series of low-complexity repeats (5’-GAATACCTGAATC-3’). BLAST nucleotide searches did not identify any complete orthologous genes within the other *Neisseria* species; the 3’ end of the gene (upstream of the repeat region) exhibited sequence similarity with non-coding regions in the genomes of *N. gonorrhoeae* and *N. lactamica*. When BLASTing the repetitive region alone, similarities with *Bacillus thuring-iensis* as well as a number of eukaryotic species (e.g. grape, horse, European polecat, and bonobo) in addition to human (Figure 3) were uncovered. NMB1848 is CpG-rich, containing more CpG dinucleotides than are expected given the underlying GC-content of *N. meningitidis*. Another gRUCP (NGTW08_1668) resulted in BLAST hits to sequences from mammalian genomes. This hit corresponds to the L1 element. The acquisition of human DNA was recently found within some strains of *N. gonorrhoeae*[61]. In this previous study, evidence of a 685bp sequence exhibiting 98-100% homology with the human L1 element was found.

Under the assumption that acquired genes will adopt a more *Neisseria*-like composition, particularly with respect to third position mutations of the codon, one can hypothesize that genes exhibiting a composition most divergent from the genome norm are relatively recent acquisitions. The converse, however, is not possible to assert; one cannot assume that those exhibiting a more *Neisseria*-like composition are older as they may have been acquired from a species exhibiting a composition similar to *Neisseria*. As the analysis of the strain-specific gRUCPs shows, a wide variety of species can be the source of horizontally acquired genes. Genes such as phage associated proteins, the capsule proteins, the *comE* family of proteins and other virulence-associated proteins were amongst the most divergent from their respective genome’s composition (Table 4; full listing in Additional file 14: Table S8). As this table shows, three of the most divergent genes were coding regions of the non-pathogenic *N. lactamica* strain; referring to the gRUCPs comparisons performed (Additional file 9: Table S3), these three (NLA_1460, NLA_8410 and NLA_13760) are in fact unique to *N. lactamica*, not occurring in any of the other *Neisseria* species nor producing any hits in our BLAST search suggesting that they were acquired within the *N. lactamica* lineage relatively recently.

**Table 4:** Top 10 genes exhibiting a composition most divergent from their genome’s respective norm

**Gene synonym**	**Serogroup (Acc#)**	**Protein product**	***R***^2^**Deviation**
NLA_13760	Nl (NC_014752)	Integral membrane protein	0.4139554
NLA_1460	Nl (NC_014752)	Hypothetical protein	0.4088421
NMO_1245	NmCln (NC_013016)	FrpA/C-related protein, truncated	0.4077326
NGO0622	Ng (NC_002946)	Hypothetical protein	0.4064973
NMAA_0648	NmA (NC_017512)	Hypothetical protein	0.4000368
NMBM01240355_0958	NmB (NC_017517)	Hypothetical protein	0.39973
NGO1938	Ng (NC_002946)	Hypothetical protein	0.3980348
NMAA_1127	NmA (NC_017512)	FrpA/C-like protein	0.3935624
NMA0941	NmA (NC_003116)	Hypothetical protein	0.3919223
NMO_1240	NmCln (NC_013016)	Superoxide dismutase	0.3910261

## Discussion

From our examination of the gRUCPs found to be unique to individual *Neisseria* strains, a number of putative sources, including other pathogenic bacteria, were identified suggesting that gene exchange occurs between *Neisseria* spp. and other bacterium within their microhabitat. Furthermore, gRUCPs identified within *N. meningitidis* strains were homologous to a number of other bacterium containing a capsule. The homology identified between a *N. meningitidis* gRUCP and the human genome revealed for the first time that *N. meningitidis* may be capable of integrating host DNA. The majority of the gRUCPs found to be unique to individual strains, however, did not BLAST to any known species or gene. While the evolutionary path of the *Neisseria* genus has long been a point of debate, our examination of the gRUCPs, which includes the pathogenome of *Neisseria* (Table 3), suggests that *N. meningitidis* is more closely related to the non-pathogenic *Neisseria* species than *N. gonorrhoeae*.

This study is consistent with previous analyses of the *N. meningitidis* genomes finding the species to have an open pan-genome [34]. The approach employed here permits one to easily identify those genes most divergent to the underlying composition of the genome. Genes which are unique and likely recent acquisitions for even the non-pathogenic *N. lactamica* genome were observed signifying that the three *Neisseria* species are all recipients of horizontally transferred elements post-speciation. Furthermore, evidence of strain specific and serotype specific acquisitions of genes were also identified. For instance, the *N. meningitidis* MC58 and *N. gonorrhoeae* NCCP11945 genomes have a number of unique gRUCPs, suggesting that these two strains have acquired genes unique to their individual evolutionary history.

The majority of the genes which are unique to a particular strain did not reveal statistically significant homologies with any sequenced species indicating that the source of these proteins remains unknown. Because these gRUCPs exhibited divergent nucleotide compositions, it is not likely that the genes are native to *Neisseria*. Rather, we hypothesize that they were acquired from another genus. For those gRUCPs which were found to be strain specific and homologous to genes within other sequenced bacterial genomes, one of two scenarios is possible: (1) the species identified from the BLAST search can be the source of the gene acquired by *Neisseria* or (2) both species could have acquired the gene from the same source. Looking at some of the organisms identified from the BLAST searches (Additional file 13: Table S7), one can find very probable instances of gene exchange. For instance, many of the BLAST results find similarities with sequences in *Haemophilus* spp. and *Moraxella catarrhalis*, other human pathogens.

The sequence similarity between gRUCPs and eukaryotic species was unexpected. While one of these gRUCPs has recently been discussed in the literature [61], the acquisition of human DNA was previously thought exclusive to *N. gonorrhoeae*. The BLAST result finding homology between the *N. meningitidis* gene NMB1848 and a portion of the PHD finger protein sequence PHF21B is the first report to our knowledge indicating human DNA uptake within this species. The region (195 nucleotides in length) is an annotated conserved domain, Formin Homology Region 1 characteristic of its low complexity repeats of around 12 residues [63]. This domain is not found within the genome sequences of either *N. gonorrhoeae* or *N. lactamica*. In fact, save a moderate homology with a sequence within the *B. thuringiensis* genome (Figure 3B), this domain appears to be eukaryotic in origin. The complete NMB1848 coding region of the MC58 strain is also present in other *N. meningitidis* strains, although not annotated as a coding region; therefore it was not identified as a gRUCP in the other *N. meningitidis* strains. Thus, we hypothesize that it was acquired post-speciation with *N. gonorrhoeae* and from a eukaryotic source, most likely its host. As such, the acquisition of host DNA is not exclusive to *N. gonorrhoeae*.

The recent sequencing of the non-pathogenic *N. lactamica* genome facilitates the identification of genes responsible for the pathogenicity of *N. gonorrhoeae* and *N. meningitidis*. Our analysis revealed two hypothetical proteins which are unique to the pathogenic *Neisseria*; further investigation is needed to ascertain if or how they are contributing to virulence. The gRUCPs conserved amongst all *N. meningitidis* strains and absent from *N. gonorrhoeae* and *N. lactamica* include several genes of interest, many of which are annotated as superoxide dismutase. Previous studies have found that *N. mening-itidis* uses these enzymes to neutralize the effect of reactive oxygen species within the host [64,65]. Furthermore, in a recent transcriptomic study, these genes were found to be upregulated during infection [65]. In congruence with previous literature [66], our analysis confirms the importance of metal transport proteins for *N. meningitidis* as they are conserved amongst all of the *N. meningitidis* strains examined. The presence of the PEMK-like protein within the *N. meningitidis* genomes and absent from the *N. gonorrhoeae* genomes suggests that this may also aid in the meningococcal infection, by interfering with host mRNA [67].

The composition-based method employed here was able to identify virulence genes previously identified within the pathogenic *Neisseria* spp. [3,7,28,49-54]. Included are the capsule genes, some of which are present in *N. gonorrhoeae* as well as non-pathogenic species of *Neisseria* (Additional file 5: Figure S5)*.* The presence of these *cps*-associated genes in *N. gonorrhoeae*, non-pathogenic *Neisseria* spp., as well as non-invasive meningococcal strains has led others to suggest that these genes have a biological role distinct from pathogenicity. *N. lactamica*’s capsule is antigenically similar to that of *N. meningitidis*[68]. The pilin gene was identified as a gRUCP for the non-pathogenic *N. lactamica*. Despite its important role in pathogenicity, the presence and structure of the pilin does not indicate the species’ ability to cause human disease [69]. The identification of the pilin genes as gRUCPs in the non-pathogenic as well as pathogenic *Neisseriae* suggests that selection is playing a role in shaping the composition and function of this gene cluster [70].

Because the vast majority of the RUCPs in *N. lactamica* are also present within the genomes of the pathogenic species, one may conclude that the two pathogenic species are more closely related to each other than either is with the non-pathogenic *N. lactamica*. This mirrors previous phylogenic analysis of the *Neisseria* spp. using a small set of homologous genes [46,47]. Unraveling the evolutionary history of the emergence of *N. gonorrhoeae* and *N. meningitidis* is far from trivial. Several genes were identified as acquisitions exclusive to *N. gonorrhoeae* or *N. meningitidis*. Moreover, genes that are present in one, two or three of the *N. gonorrhoeae* genomes and are present within all (or the majority) of the *N. meningitidis* strains examined here suggest their acquisition prior to the divergence of the two species. More baffling are the instances in which a gene is present in a single *N. gonorrhoeae* genome and in just a few (and different serotypes) of the *N. meningitidis* strains. Looking specifically at the gRUCPs present within all 18 genomes considered, a stronger similarity between *N. meningitidis* and *N. lactamica* is observed. If these gRUCPs were acquired prior to speciation of *N. lactamica* and the pathogenic species, this would suggest that *N. meningitidis* is more closely related to the non-pathogenic *N. lactamica*[40,48,49]. The availability of signifi-cantly more *N. meningitidis* genomes, however, is likely contributing to this observation. Further sequencing and annotation of *N. gonorrhoeae* genomes as well as additional *Neisseria* spp. is underway and will likely shed further light on the evolution of the genus.

## Conclusions

The approach employed here presents a new means by which investigators can readily identify genes unique to a species or strain as well as identify genes which exhibit compositional properties aberrant from the genome norm such as acquired elements and genes under unique selective pressures. Analysis of the identified GEIs revealed that gene exchange occurs frequently between *Neisseria* spp. and other bacteria within their microhabitat; it also occurs with their human host. From the genic material acquired by the 18 strains examined here, albeit not a comprehensive representation of the strains present in nature, it appears that the *N. meningitidis* strains are more closely related to the non-pathogenic *Neisseria lactamica* than the *N. gonorrhoeae* strains are.

The genes identified as exclusive to the pathogenic *Neisseria* genomes, *N. meningitidis* genomes, or *N. gonor-rhoeae* genomes provide insight into the genus both from an evolutionary perspective as well as from the perspective of that of a microbiologist. Given the prevalence of the pathogenic species, identifying additional factors which aid in the virulence of *Neisseria* is of importance. Genes involved in iron and zinc uptake and downstream processes have been thoroughly investigated as candidates for the development of vaccines (e.g. [71,72]). Included in the RUCPs identified here are a number of hypothetical proteins which warrant further investigation as putative virulence factors.

## Methods

### Neisseria genome sequences

The complete genomes of three *N. gonorrhoeae*, 14 *N. meningitidis*, and one *N. lactamica* strains were obtained from NCBI’s FTP site. All of these genomes have been assembled and annotated [34,36,43,54,55,73-79]. The annotation files for all these genomes were collected (*.ptt and *.rnt files). Table 1 lists the genomes.

### Quantifying genomic similarity and dissimilarity

The compositional profile of each window, in the case of the sliding window approach, or each gene, in the case of the gene-by-gene approach, was computed as previously described [37] and summarized as follows. Looking at an individual window or gene sequence, the frequency of occurrence of all *k*-mers is determined. When evaluating the similarity/dissimilarity of two windows or two genes, these two frequency profiles can be compared. While a variety of different methods can be implemented, here we quantify distance according to the coefficient of determination (*R*^2^). The distribution *P*(*S*) of appearances of all possible *k*-mers inside a given window is *P*(*S*) = *N*_*S*_/(*w* − *k* + 1), where *N*_*S*_ and *w* are, correspondingly, the number of appearances of *k*-mer *S* and the total number of *k*-mers in the window being examined. The correlation statistic is appropriate only when representative statistics are collected such that *N*_*s*_ > 1; therefore one must impose the condition *w* > 4^*k*^ when taking either the sliding window or gene-by-gene approach.

Analyses were conducted for a variety of different sizes of *k* meeting the condition *w* > 4^*k*^. For the window-by-window analysis with a window size of 5000 nucleotides, *k* = 6 was used. wRUCPs identified for this *k*-mer size were also found when *k* = 5 and *k* = 4. Because the length of genes varies across the genome, the gene-by-gene analysis necessitated a choice of *k* such that 4^*k*^ is smaller than the shortest annotated coding region. As such, the largest size *k* which can be used is 3. Because our analysis focuses on 3-mer and 6-mer compositional profiles (capturing the underlying codon usages), genes which do not exhibit the *Neisseria* codon usage and are likely from foreign sources are identified as exhibiting an unusual composition.

To assess the similarity of the compositional properties of a particular window *i* within its genomic sequence, the average *R*^2^ value as well as the standard deviation is calculated. Thus, windows which exhibit the same *k*-mer usage profiles will have a *R*^2^ value approaching 1. Windows containing regions which have not been under strong selection to follow the genome-wide composition (e.g. mono- or dinucleotide biases or codon biases), such as rRNA, will be less similar to the other windows in its genome, thus resulting in a smaller *R*^2^ value. Likewise, foreign DNA recently integrated into the genome, e.g. horizontally transferred elements, will also be less similar to the other windows in its genome. As a threshold, we select those windows/genes having an *R*^2^ value two standard deviations from the genome average *R*^2^ value as exhibiting unusual compositional properties. Of the 18 genomes examined here, no windows/genes had an *R*^2^ value greater than or equal to two standard deviations greater than the genome average. Given our interest in regions acquired from foreign sources, putative virulence factors and genes under strong selection (despite genome compositional proclivities), genes such as rRNA were not classified as RUCPs. We refer to the regions exhibiting unusual compositional properties from the window-by-window analysis as wRUCPs and from the gene-by-gene analysis gRUCPs.

The source code, including fixed and variable window functionality implemented in C++, is freely available upon request.

### Visualizing similarity and dissimilarity between genomes

To visualize the similarity, we plot the matrix of correlation coefficients (*r*), *C*(*i,j*), between the distributions of *k*-mers, where *i* is a window in the first genome and *j* is a window in the second genome. (Note, visualization is performed using *r* values not *R*^*2*^ values in an effort to expand the spectrum of values observed). The vertical and horizontal coordinates in an S-plot represent the location of windows *i* and *j*, respectively. Different correlation coefficients are represented on the plots by different colors. An application to generate S-Plots using the C# language for Windows was created and is freely available at http://www.bioinfo.uh.edu/splot. For further details, the reader is referred to [37] and the online documentation accompanying the application. The gene-by-gene functionality is not included within the visualization tool.

### Comparative analysis of RUCPs

wRUCPs and gRUCPs were firstly compared to the other members of the *Neisseria* spp. as well as all publicly available genomes. Similarities between *Neisseria* spp. were conducted by examining correlations between each individual RUCP and all windows in the other *Neisseria* sequences. Sequences were either aligned using BioEdit (http://www.mbio.ncsu.edu/bioedit/bioedit.html) or BLASTed. BLAST-based analysis was performed as follows. For each RUCP, be it a window or an individual gene, the sequence was parsed from the complete genome. Using the BLAST web interface, each sequence was compared to all sequences within the nucleotide collection nr/nt database using BLASTn with a word size of 7. In addition, the search was conducted with the exclusion of the taxonomical group for *Neisseria* (taxid: 482) and an e-value threshold of 0.5. Upon inspection of the results for each sequence, only those hits meeting the aforementioned threshold and resulting in a query coverage greater than 50% were considered homologous. Genes which uniquely appeared in a single *Neisseria* strain were also compared to all publicly available genomes in the nucleotide collection nr/nt database. The same threshold, e-value and exclusion as our previous BLAST analysis were applied again here. BLASTx searches were also conducted to examine the similarity of gRUCPs conserved amongst all pathogenic *Neisseria* spp., amongst all *N. meningitidis* spp., and amongst all *N. gonorrhoeae* spp. with other *Neisseria* sequences which were not included in this analysis as well as other bacterial genomes. A word size of 2 was used for more sensitive searches. All e-values less than 1.0 were considered putative hits.

## Competing interests

The authors declare that they have no competing interests.

## Authors’ contributions

CP, BN, SN, and YF designed the study. CP and YF developed the software. MS and CP carried out the bioinformatic analysis. All authors participated in the drafting of the manuscript as well as read and approved the final manuscript.

## Supplementary Material

Additional file 1: Figure S1 The S-plot of *N. meningitidis* MC58 (Serogroup B) vs. *N. meningitidis* Z2491 (Serogroup A). Click here for file

Additional file 2: Figure S2 The S-plot of *N. meningitidis* Z2491 (Serogroup A) vs. *N. gonorrhoeae* FA 1090. Click here for file

Additional file 3: Figure S3 The S-plot of *N. meningitidis* Z2491 (Serogroup A) vs. *N. lactamica* ST-640. Click here for file

Additional file 4: Figure S4 The S-plot of *N. gonorrhoeae* FA 1090 vs. *N. lactamica* ST-640. Click here for file

Additional file 5: Figure S5 Compares the *N. meningitidis* serogroup C strain FAM18 with (a) an *N. meningitidis* serogroup A strain, (b) an *N. gonorrhoeae* strain, and (c) the *N. lactamica* strain, highlighting the location of genes associated with the capsule. Click here for file

Additional file 6: Table S1 Includes the complete listing of the wRUCPs identified in the 18 *Neisseria* strains. Click here for file

Additional file 7: Table S2 Includes the complete listing of the gRUCPs identified in the 18 *Neisseria* strains. Click here for file

Additional file 8: Figure S6 Illustrates the *R*^2^ value for each gRUCPs identified in the serogroup A *N. meningitidis* Z2491 strain and the closest coding region found within in each of the other 17 genomes. Click here for file

Additional file 9: Table S3 Lists the maximum homology score (*R*^2^) to genes within other *Neisseria* strains for each gRUCP. Click here for file

Additional file 10: Table S4 The list of genes identified as gRUCPs which were conserved amongst all pathogenic *Neisseria* strains and absent from the *N. lactamica* ST-640 genome sequence. Carrier strains were excluded from analysis. Click here for file

Additional file 11: Table S5 Lists the genes identified as gRUCPs which were conserved amongst all *N. meningitidis* strains but are not present in any of the examined *N. gonorrhoeae* strains or the *N. lactamica* ST-640 genome sequence. Carrier strains were excluded from analysis. Click here for file

Additional file 12: Table S6 The list of genes identified as gRUCPs which were conserved amongst all *N. gonorrhoeae* strains and not present in any of the examined *N. meningitidis* strains or the *N. lactamica* ST-640 genome sequence. Carrier strains were excluded from analysis. Click here for file

Additional file 13: Table S7 Presents the BLASTn results for the 217 genes which appear to be unique to a single *Neisseria* strain. Click here for file

Additional file 14: Table S8 Provides information regarding the genes exhibiting a composition most divergent from the genome norm. Click here for file
