# Supplementary material for: Where does Neisseria acquire foreign DNA from: an examination of the source of genomic and pathogenic islands and the evolution of the Neisseria genus
Source: BMC Evol Biol. 2013 Sep 4;13:184. doi: 10.1186/1471-2148-13-184 (PMC3848584; doi:10.1186/1471-2148-13-184)
Supplement: Additional file 1: Figure S1 — The S-plot of N. meningitidis MC58 (Serogroup B) vs. N. meningitidis Z2491 (Serogroup A). [file 1471-2148-13-184-S1.pdf]

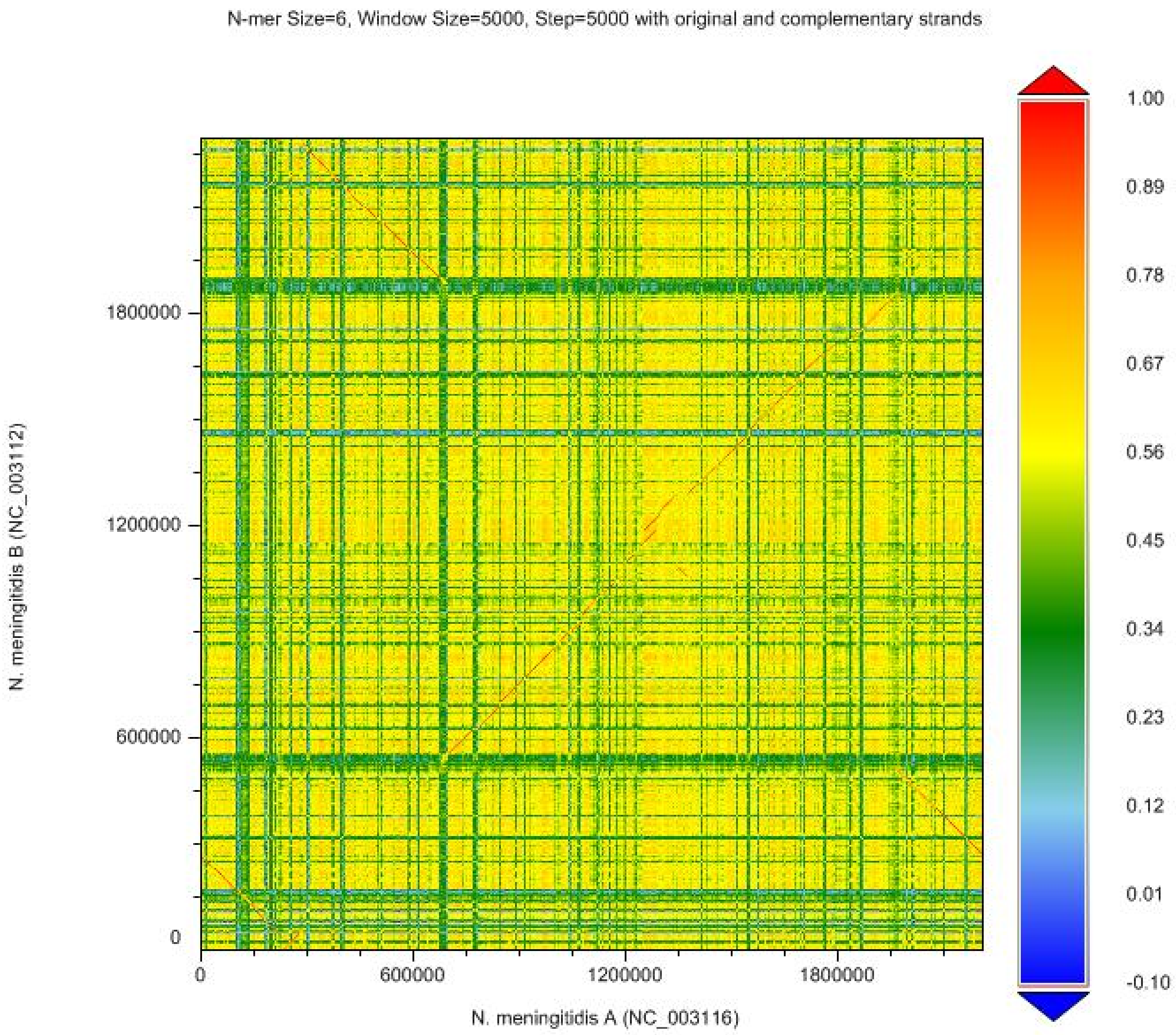

N-mer Size=6, Window Size=5000, Step=5000 with original and complementary strands
N. meningitidis B (NC_003112)
1800000
1200000
600000
0
0
600000
1200000
1800000
N. meningitidis A (NC_003116)
1.00
0.89
0.78
0.67
0.56
0.45
0.34
0.23
0.12
0.01
-0.10
