# Supplementary figures and images for: Where does Neisseria acquire foreign DNA from: an examination of the source of genomic and pathogenic islands and the evolution of the Neisseria genus
Source: BMC Evol Biol. 2013 Sep 4;13:184. doi: 10.1186/1471-2148-13-184 (PMC3848584; doi:10.1186/1471-2148-13-184)

N-mer Size=6, Window Size=5000, Step=5000 with original and complementary strands

NC\_003116

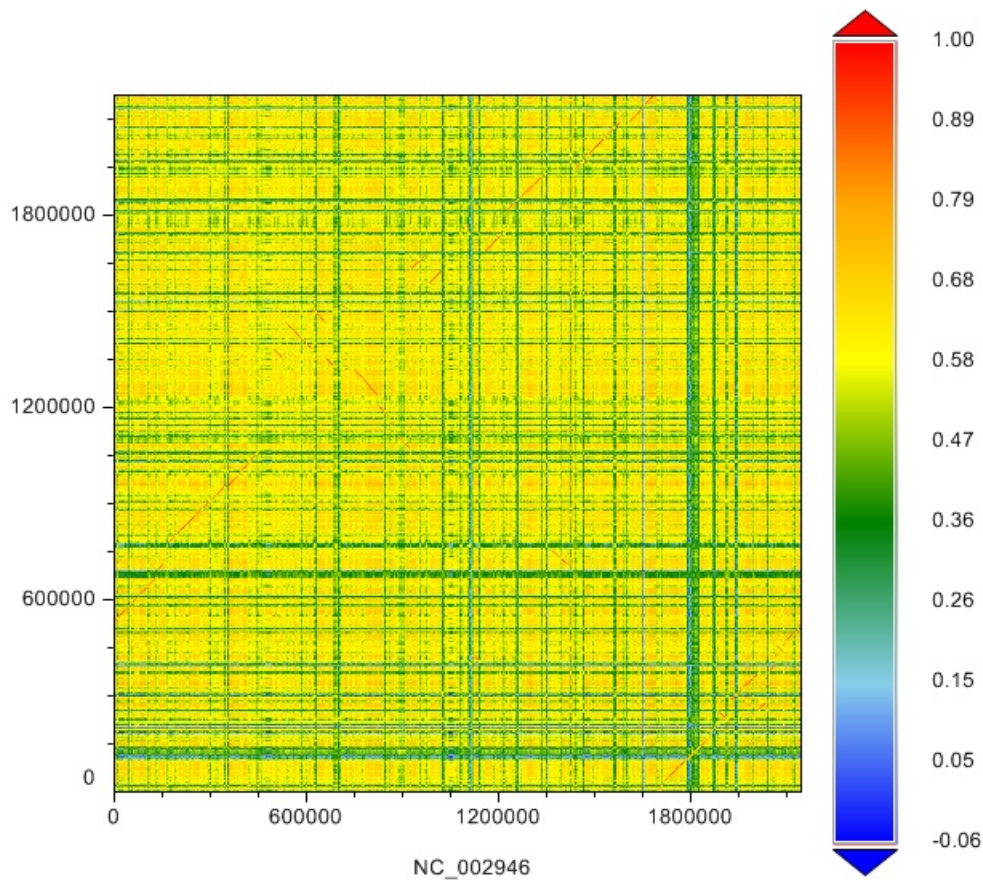

Supplement: Additional file 2: Figure S2 — The S-plot of N. meningitidis Z2491 (Serogroup A) vs. N. gonorrhoeae FA 1090. [file 1471-2148-13-184-S2.pdf]

N-mer Size=6, Window Size=5000, Step=5000 with original and complementary strands

NC\_003116

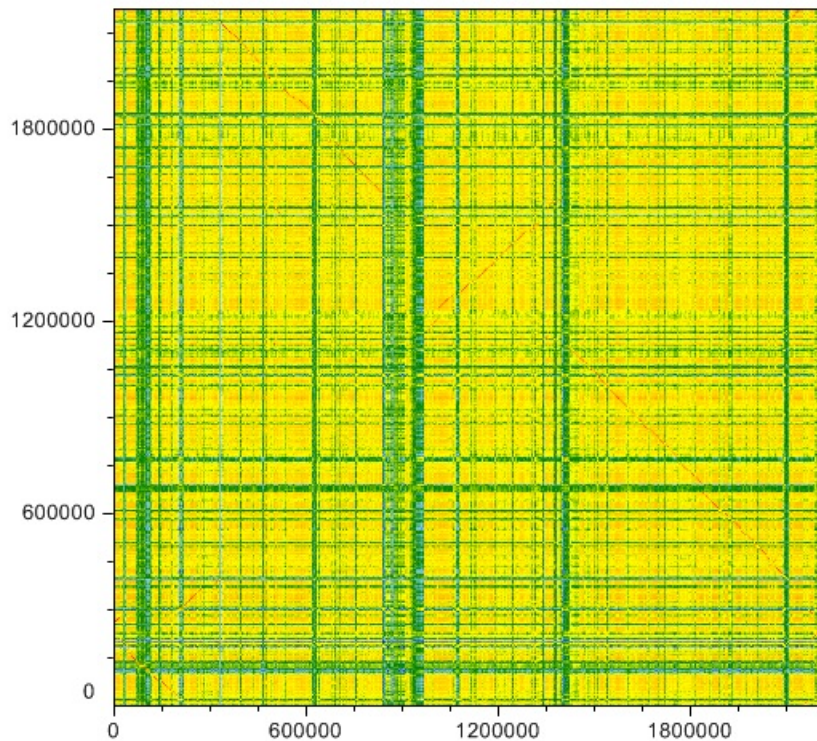

NC\_014752

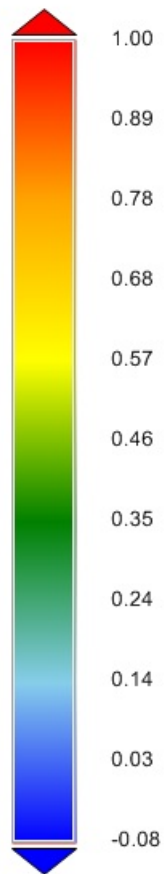

Supplement: Additional file 3: Figure S3 — The S-plot of N. meningitidis Z2491 (Serogroup A) vs. N. lactamica ST-640. [file 1471-2148-13-184-S3.pdf]

N-mer Size=6, Window Size=5000, Step=5000 with original and complementary strands

NC\_002946

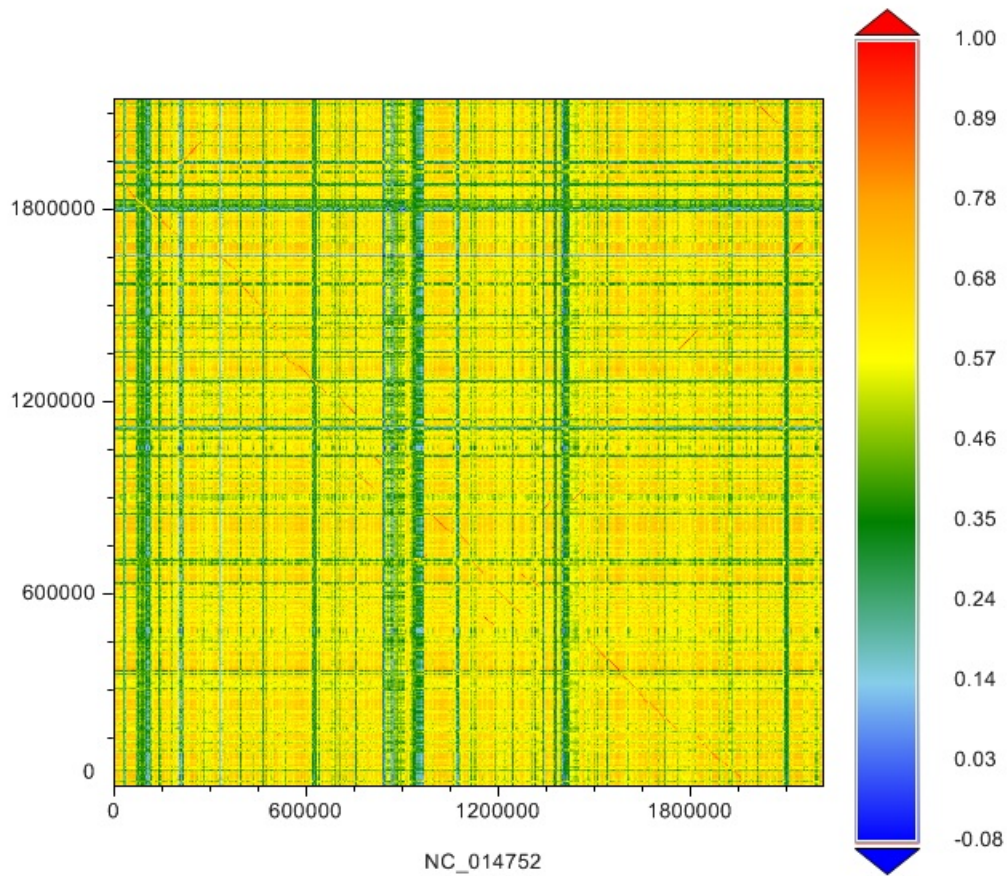

Supplement: Additional file 4: Figure S4 — The S-plot of N. gonorrhoeae FA 1090 vs. N. lactamica ST-640. [file 1471-2148-13-184-S4.pdf]

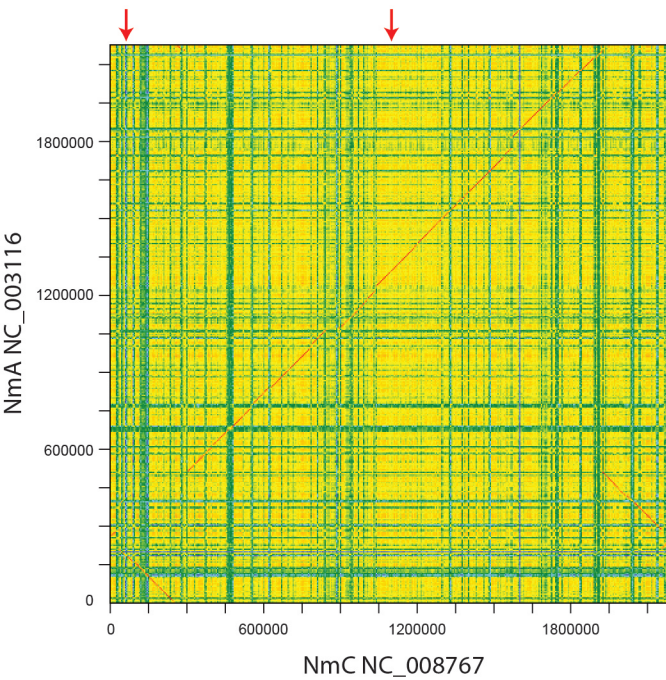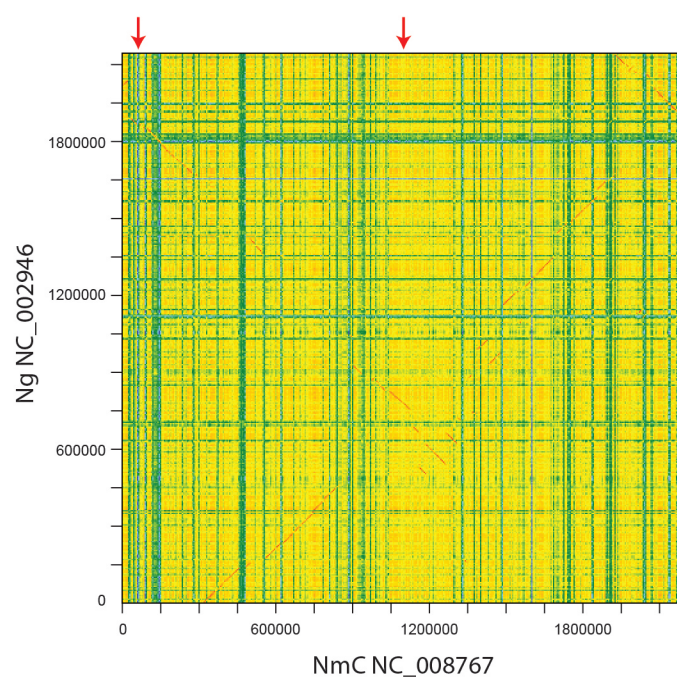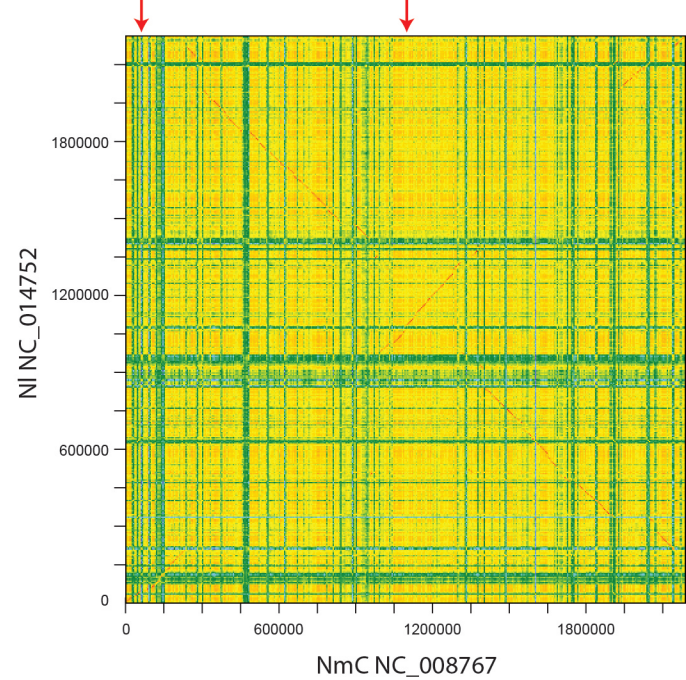

Supplement: Additional file 5: Figure S5 — Compares the N. meningitidis serogroup C strain FAM18 with (a) an N. meningitidis serogroup A strain, (b) an N. gonorrhoeae strain, and (c) the N. lactamica strain, highlighting the location of genes associated with the capsule. [file 1471-2148-13-184-S5.pdf]

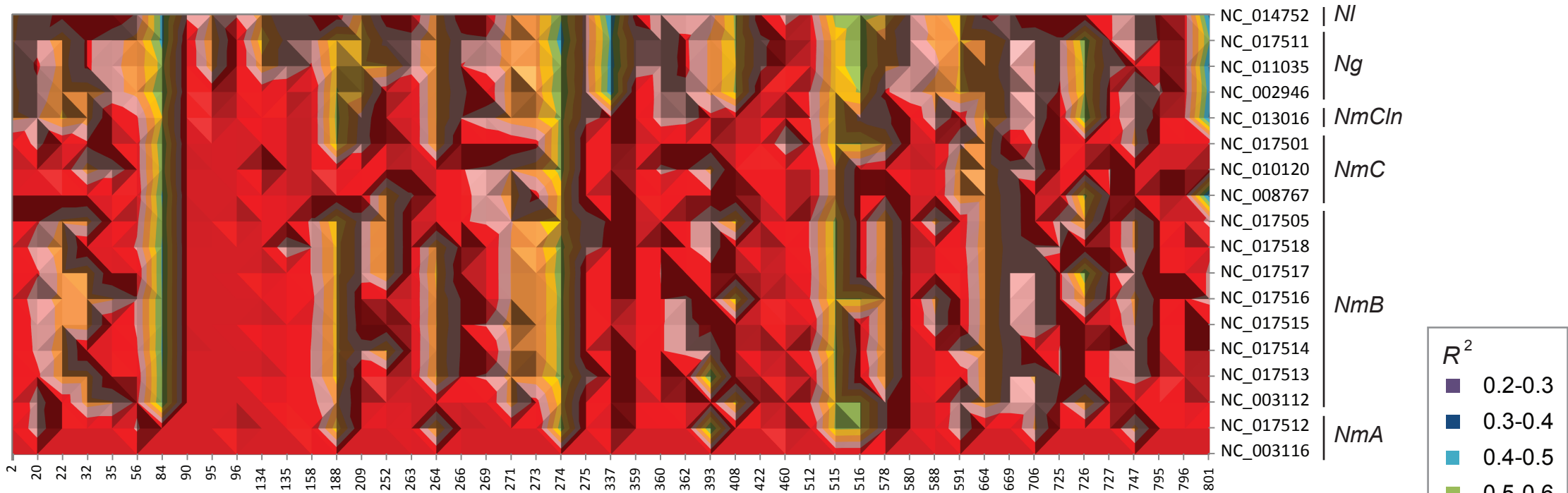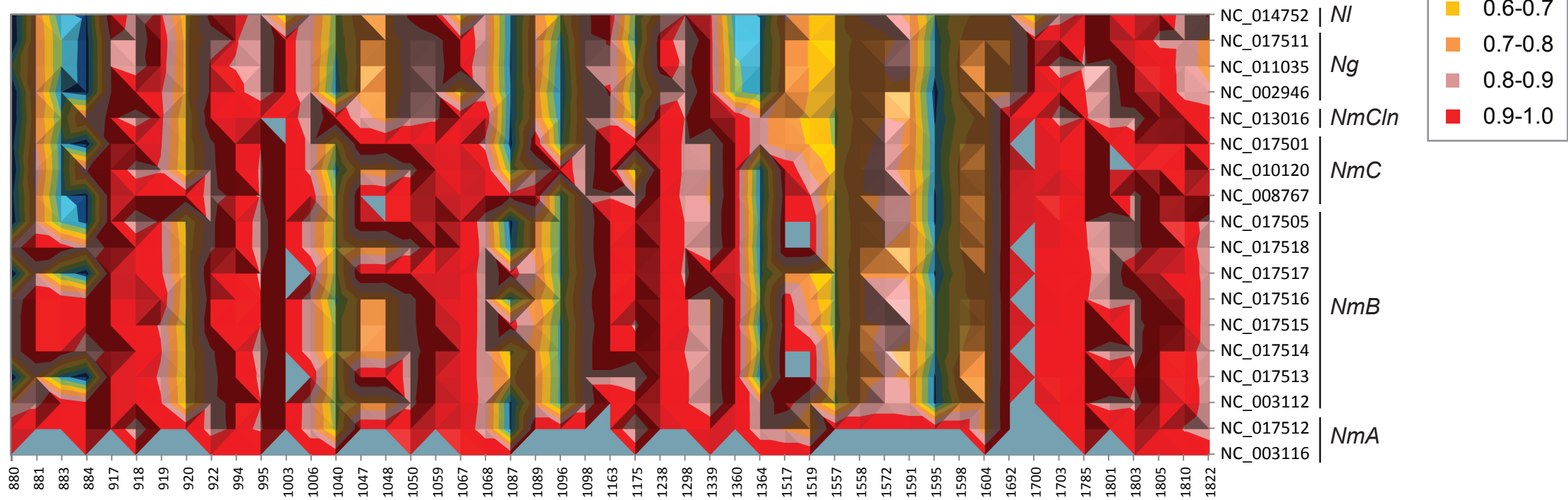

Supplement: Additional file 8: Figure S6 — Illustrates the R2 value for each gRUCPs identified in the serogroup A N. meningitidis Z2491 strain and the closest coding region found within in each of the other 17 genomes. [file 1471-2148-13-184-S8.pdf]
