# Supplementary material for: Where does Neisseria acquire foreign DNA from: an examination of the source of genomic and pathogenic islands and the evolution of the Neisseria genus
Source: BMC Evol Biol. 2013 Sep 4;13:184. doi: 10.1186/1471-2148-13-184 (PMC3848584; doi:10.1186/1471-2148-13-184)
Supplement: Additional file 10: Table S4 — The list of genes identified as gRUCPs which were conserved amongst all pathogenic Neisseria strains and absent from the N. lactamica ST-640 genome sequence. Carrier strains were excluded from analysis. [file 1471-2148-13-184-S10.pdf]

Table S4. Genes identified as gRUCPs which were conserved amongst all pathogenic *Neisseria* strains and absent from the *N. lactamica* ST-640 genome sequence. Carrier strains were excluded from analysis.

Reference Gene Sequence	***Protein Functionality***	***BLASTn and BLASTx Analysis***
NMA0103	DNA-directed RNA polymerase subunit alpha	Present in the genome sequence of *N. lactamica* 020-06 but not the *N. lactamica* complete, annotated RefSeq genome examined here.
NMA0109	preprotein translocase subunit SecY	Present in the genome sequence of *N. lactamica* 020-06 but not the *N. lactamica* complete, annotated RefSeq genome examined here.
NMA1169	hypothetical protein	Annotated in some spp as "phage associated protein". Present also in *Burkholderia* spp.
NMBG2136_0520	hypothetical protein	Annotated in some spp as a "putative membrane protein". Not found in any other bacterial spp.
NGO0163	hypothetical protein	Not found in any other bacterial spp.
NGO0449	hypothetical protein	Annotated as a ribonuclease T (rnt) in other bacterial spp.
NGO1818	DNA-directed RNA polymerase subunit alpha	Present in the genome sequence of *N. lactamica* 020-06 but not the *N. lactamica* complete, annotated RefSeq genome examined here.
NGO2145	ATP synthase F0F1 subunit C	Present in the genome sequence of *N. lactamica* 020-06 but not the *N. lactamica* complete, annotated RefSeq genome examined here.
