# Supplementary material for: Where does Neisseria acquire foreign DNA from: an examination of the source of genomic and pathogenic islands and the evolution of the Neisseria genus
Source: BMC Evol Biol. 2013 Sep 4;13:184. doi: 10.1186/1471-2148-13-184 (PMC3848584; doi:10.1186/1471-2148-13-184)
Supplement: Additional file 11: Table S5 — Lists the genes identified as gRUCPs which were conserved amongst all N. meningitidis strains but are not present in any of the examined N. gonorrhoeae strains or the N. lactamica ST-640 genome sequence. Carrier strains were excluded from analysis. [file 1471-2148-13-184-S11.pdf]

Table S5. Genes identified as gRUCPs which were conserved amongst all *N. meningitidis* strains but are not present in any of the examined *N. gonorrhoeae* strains or the *N. lactamica* ST-640 genome sequence. Carrier strains were excluded from analysis.

Reference Gene Sequence	***Protein Functionality***	***BLASTn and BLASTx Analysis***
NMA0003	hypothetical protein	Found in other bacterial spp. While not found in *N. gonorrhoeae*, it is present in the *N. lactamica* ATCC 23970 but not the *N. lactamica* complete, annotated RefSeq genome examined here.
NMA0400	PEMK-like protein	Found in other bacterial spp.
NMA0685	hypothetical protein	Annotated in some *N. meningitidis* spp as a "putative membrane protein". Not found in any other bacterial spp.
NMA1617	superoxide dismutase	Found in other bacterial spp.
NMA1988	integral membrane protein	Annotated in other bacterial spp. as a "heavy metal transport protein".
NMAA_0355	hypothetical protein	Annotated in some spp as a "putative membrane protein". Not found in any other bacterial spp.
NMAA_1445	putative heavy metal transport protein	Found in other bacterial spp.
NMBG2136_0160	hypothetical protein	Found in *N. gonorrhoeae* spp. but is not annotated as a coding region for BLASTn. No significant similarity found for BLASTx analysis (word size=2).
NMBM01240149_0758	hypothetical protein	Annotated in other bacterial spp. as a "superoxide dismutase".
NMBM01240355_2021	adenine-specific methyltransferase HpaI	Found in other bacterial spp.
NMC1806	hypothetical protein	Annotated in some *N. meningitidis* spp as the "FrpC operon protein". Not found in any other bacterial spp.
