# Supplementary material for: Where does Neisseria acquire foreign DNA from: an examination of the source of genomic and pathogenic islands and the evolution of the Neisseria genus
Source: BMC Evol Biol. 2013 Sep 4;13:184. doi: 10.1186/1471-2148-13-184 (PMC3848584; doi:10.1186/1471-2148-13-184)
Supplement: Additional file 12: Table S6 — The list of genes identified as gRUCPs which were conserved amongst all N. gonorrhoeae strains and not present in any of the examined N. meningitidis strains or the N. lactamica ST-640 genome sequence. Carrier strains were excluded from analysis. [file 1471-2148-13-184-S12.pdf]

Table S6. Genes identified as gRUCPs which were conserved amongst all *N. gonorrhoeae* strains and not present in any of the examined *N. meningitidis* strains or the *N. lactamica* ST-640 genome sequence. Carrier strains were excluded from analysis.

Reference Gene Sequence	***Protein Functionality***	***BLASTn and BLASTx Analysis***
NGO0302	hypothetical protein	Present only in the *N. gonorrhoeae* sequences and *N. polysaccharea*.
NGO0355	hypothetical protein	Found in other bacterial spp., some annotated as a "putative transposase". While not found in *N. meningitidis*, it is present in the *N. lactamica* ATCC 23970 but not the *N. lactamica* complete, annotated RefSeq genome examined here.
NGO0466	phage associated protein	Not found in any other bacterial spp.
NGO0476	phage associated protein	Not found in any other bacterial spp.
NGO0784	hypothetical protein	Not found in any other bacterial spp.
NGO0865	hypothetical protein	Found in *N. meningitidis* spp. but is not annotated as a coding region for BLASTn. Also found in *Moraxella catarrhalis* which colonizes in the human nasal passageway.
NGO1010	phage associated protein	Not found in any other bacterial spp.
NGO1147	hypothetical protein	Although BLASTn shows no similarity to other *Neisseria* spp., BLASTx reveals sequence similarity at the protein level with *N. lactamica* ATCC 23970 as well as *N. meningidis* 69166 (neither of which have a complete, annotated RefSeq genome and thus were not examined here) in addition to a strain of *N. flavescens*.
NGO1296	hypothetical protein	Found in *N. meningitidis* spp. as well as *N. lactamica* 020-06 but is not annotated as a coding region for BLASTn.
NGO1445	hypothetical protein	BLASTn reveals presence in *N. lactamica* 020-06 strain but not the *N. lactamica* complete, annotated RefSeq genome examined here. BLASTx found partial (~60%) similarity to annotated "adhesion" and "hemagluttinin-like protein" within other bacterial spp.
NGO1632	phage associated protein	Not found in any other bacterial spp.
NGO1653	hypothetical protein	BLASTx shows partial (<50%) similarity to sequences within *N. meningitidis, N. lactamica, N. mucosa, N. flavescens,* and *N. polysaccharea.*
NGO2086	hypothetical protein	Annotated in some *N. gonorrhoeae* strains as a "cell surface protein". Although BLASTn shows no similarity to other *Neisseria* spp., BLASTx reveals sequence similarity at the protein level with several *N. lactamica* strains but not the complete, annotated RefSeq genome examined here as well as a strain of *N. flavescens*.
